# An efficient magnetic resonance image data quality screening dashboard

**DOI:** 10.1002/acm2.13557

**Published:** 2022-02-11

**Authors:** Evan D. H. Gates, Adrian Celaya, Dima Suki, Dawid Schellingerhout, David Fuentes

**Affiliations:** ^1^ Department of Imaging Physics MD Anderson Cancer Center The University of Texas Houston Texas USA; ^2^ Department of Neurosurgery MD Anderson Cancer Center The University of Texas Houston Texas USA; ^3^ Departments of Cancer Systems Imaging and Neuroradiology MD Anderson Cancer Center The University of Texas Houston Texas USA; ^4^ MD Anderson Cancer Center UTHealth Graduate School of Biomedical Sciences Houston Texas USA

**Keywords:** dashboard, data curation, imaging informatics, MRI

## Abstract

**Purpose:**

Complex data processing and curation for artificial intelligence applications rely on high‐quality data sets for training and analysis. Manually reviewing images and their associated annotations is a very laborious task and existing quality control tools for data review are generally limited to raw images only. The purpose of this work was to develop an imaging informatics dashboard for the easy and fast review of processed magnetic resonance (MR) imaging data sets; we demonstrated its ability in a large‐scale data review.

**Methods:**

We developed a custom R Shiny dashboard that displays key static snapshots of each imaging study and its annotations. A graphical interface allows the structured entry of review data and download of tabulated review results. We evaluated the dashboard using two large data sets: 1380 processed MR imaging studies from our institution and 285 studies from the 2018 MICCAI Brain Tumor Segmentation Challenge (BraTS).

**Results:**

Studies were reviewed at an average rate of 100/h using the dashboard, 10 times faster than using existing data viewers. For data from our institution, 1181 of the 1380 (86%) studies were of acceptable quality. The most commonly identified failure modes were tumor segmentation (9.6% of cases) and image registration (4.6% of cases). Tumor segmentation without visible errors on the dashboard had much better agreement with reference tumor volume measurements (root‐mean‐square error 12.2 cm^3^) than did segmentations with minor errors (20.5 cm^3^) or failed segmentations (27.4 cm^3^). In the BraTS data, 242 of 285 (85%) studies were acceptable quality after processing. Among the 43 cases that failed review, 14 had unacceptable raw image quality.

**Conclusion:**

Our dashboard provides a fast, effective tool for reviewing complex processed MR imaging data sets. It is freely available for download at https://github.com/EGates1/MRDQED.

## 1 INTRODUCTION

Professional organizations like the American Association of Physicists in Medicine and the Radiological Society of North America have recently identified systematic curation of high‐quality image data sets as a key rate‐limiting step in the development of artificial intelligence in medicine.^1^, ^2^ While public repositories like The Cancer Imaging Archive^3^, ^4^ provide excellent resources, evaluating three‐dimensional (3D) image data sets and corresponding annotations in new data is still a labor‐intensive task. This is especially true in a research setting where complex data processing is used for radiomics or deep learning analysis^5^ and existing software is not well suited to viewing imaging studies in combination or rapid succession.

Some quality‐control tools have been developed for magnetic resonance (MR) image data sets. But, they focus almost exclusively on the quality and statistics of the raw images themselves. They do not help with the review of other facets of the data, such as registrations or segmentations, which are critical to quantitative measurements. Thus, there is a need for a single tool for both systematically reviewing image quality and checking the accuracy of the derived images and measurements. The goal of this project was to develop a review interface for MR data sets that had several key characteristics:

- Minimal development time and no requirement for extensive programming or information technology resources
- No requirement for specialized skills or knowledge
- Remote accessibility
- Fast loading and processing of data
- A comprehensive review of raw data, derived data, and annotations
- Integrated, persistent, structured methods to record and share reviews

Our solution was to develop a dashboard to quickly and efficiently visualize all of the necessary image data for a single case and record the quality of the various images, masks, etc. that affect the final image measurements. We rendered a handful of key slices as static portable network graphics (PNG) image files as part of the data processing pipeline: essentially, we front‐loaded the cost to load the data at the expense of the ability to freely scroll through image slices. Reviewing just a few orthogonal slices through segmentation or image was generally sufficient to screen for unacceptable data quality.

We used the dashboard to efficiently review 1380 brain tumor imaging studies that were processed as part of a large‐scale research study. In addition to calculating the various failure rates in our data‐processing pipeline, we identified which studies had acceptable data quality, which contained minor errors, and which should be excluded from further analysis. Using this classification, we compared the segmented tumor volume with the reference measurements to show how higher quality segmentations have smaller average errors. We also tested our dashboard using 2018 MICCAI Brain Tumor Segmentation Challenge (BraTS) data (285 studies).

## 2 METHODS

We implemented a dashboard interface that systematically displays representative slices of each image and segmentation using the R Shiny package.* Shiny solves many of the challenges of implementing a dashboard, such as a user interface, HTML, hosting, and reactive programming. A simplified and broadly applicable version of the app is freely available at https://github.com/EGates1/MRDQED. The dashboard code is flexible enough to use for most projects involving 3D image datasets with annotations saved in NIfTI format.

The app loads a data file with case IDs, image file paths, and any other desired tabular data. Before the app runs, the images and segmentation are used to generate key PNG snapshots. We rendered PNGs of axial, sagittal, and coronal slices for each image, with each segmentation overlaid as part of our existing data processing pipeline. The slice displayed was the one with the maximum area in the segmentation. As part of this process, the various segmentations (brain mask, three‐label tumor segmentation, and cerebral spinal fluid [CSF] ROI) were loaded together and assigned separate distinct colors. We also rendered scaled density plots of the combined segmentation, as well as three‐plane crosshair views centered on the maximum intensity voxel within the tumor segmentation for each image. By rendering snapshots as part of the initial data processing (and not during data review), the computational time is effectively front‐loaded so that all the dashboard does is render the existing PNGs.

**FIGURE 1:**
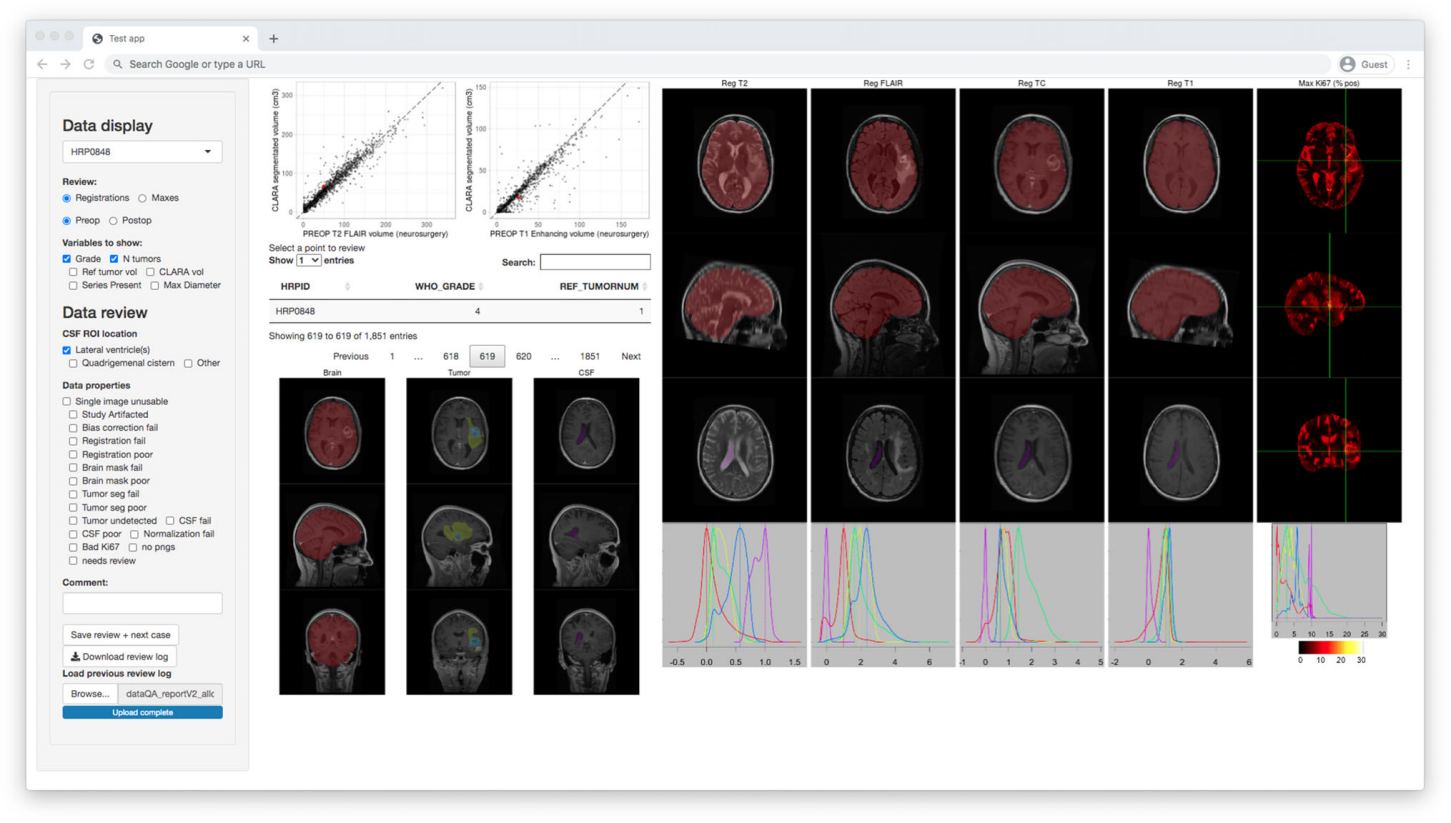
Screenshot of the data review app interfaces captured at 1440 × 900 pixels display resolution. The app is accessed via a web browser from anywhere on the institutional network

A screenshot of the app is shown in Figure 1. The dashboard consists of two main sections: a data review panel to select cases and input the results of the review and a data display area that displays PNGs of the study images and segmentations. The top portion of the data review panel has a drop‐down menu to select cases by ID and checkboxes to add data to the table. The bottom of the data review panel is used to evaluate the current case. A series of boxes is used to mark the location of the CSF ROI, which was a project‐specific feature. Below that, checkboxes are used to identify issues with the specific data. For the brain mask, tumor segmentation, or CSF ROI, the “poor” box designates minor errors that will probably not affect downstream data processing, whereas the “fail” box indicates an unusable segmentation that will interfere with later processing steps. A few other specific boxes are:

- Study artifact: All images in the study have large artifacts, are corrupted or are otherwise not fit for further data processing.
- Image unusable: One or more images are not fit for further data processing.
- Bias field: Either a strong visible bias field is visible on one or more images or bias correction was applied that has corrupted the intensities.
- Normalization failure: The intensity range on the image histograms is unreasonably outside of the expected range.
- No PNGs: Some PNGs are missing or not displayed in the app.
- Needs review: Closer inspection of the study is necessary outside of the dashboard.

Below the checkboxes is a free text comment box to add additional review notes. After reviewing a case, the “save + next” button loads the next case to review in 3–5 s. There is also a download button that exports a comma‐separated values file of the reviews entered via the current web browser. R Shiny supports multiple concurrent user sessions but does not save data between sessions. Thus, each time a reviewer connects, they can upload the latest review log to re‐populate the fields and continue.

**FIGURE 2:**
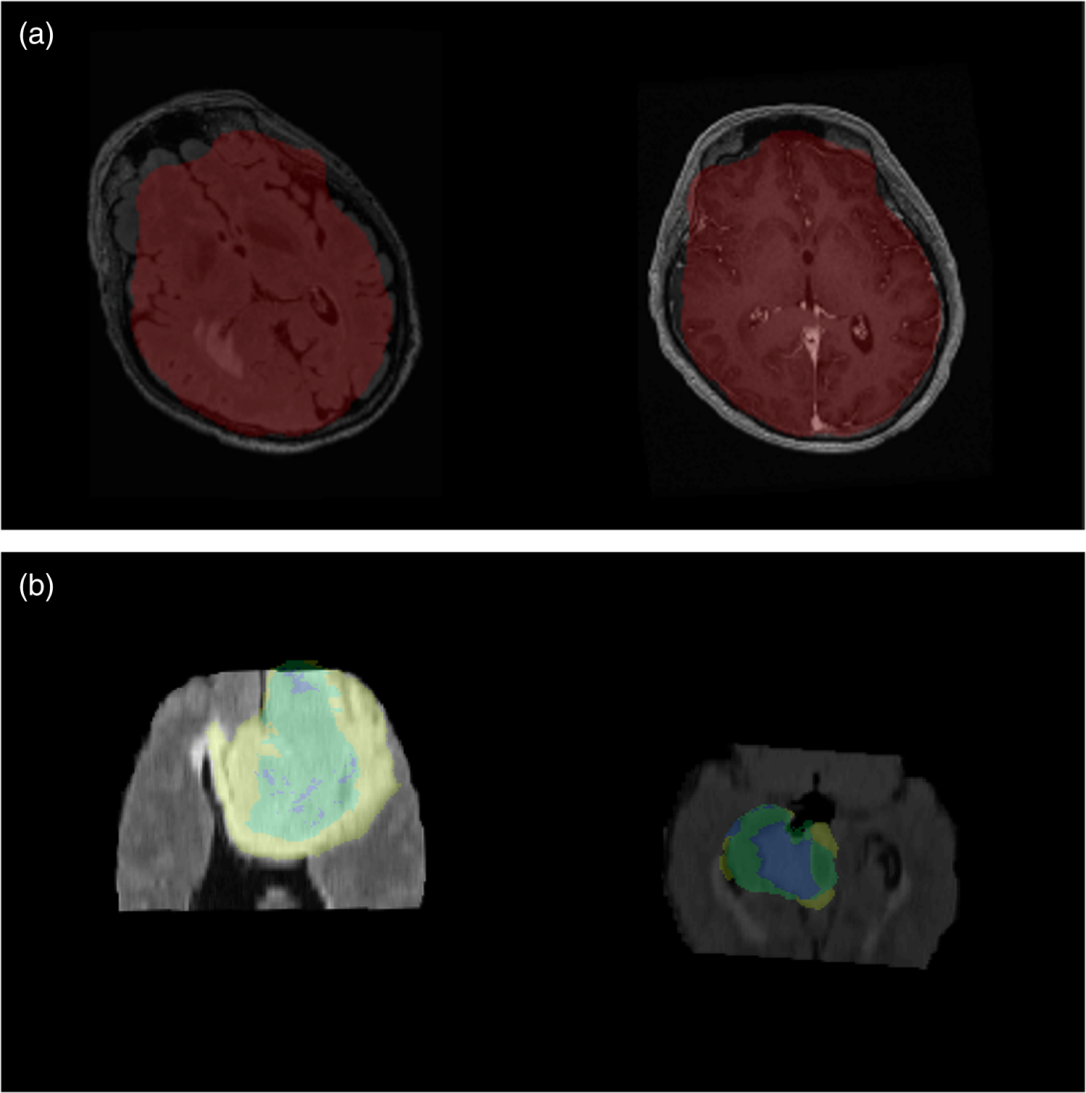
(a) Example of a failed registration identified by the dashboard. By overlaying the same brain mask on both images the relative rotation of the fluid‐attenuated inversion recovery (FLAIR) image (left) relative to the T1‐weighted image (right) is identified. Note, minor errors in the brain mask can also be seen. (b) FLAIR images and ground truth tumor segmentations included in the 2018 Brain Tumor Segmentation Challenge. Left: Brats_2013_0_1. Right: Brats18_2013_6_1. In both cases, the image field of view is so short that the segmentation is partially outside the brain volume. Both of these were caught by data review

The top left of the data display panel (Figure 1) displays scatter plots of segmented tumor volume and a project‐specific reference tumor volume.† The currently opened case is highlighted on the plot to determine how well it agrees and aligns with the population. Individual points (e.g., outliers) are also clickable to be opened for review. Below the scatter plots, a data table displays any data in the app data file that are not image file paths. The table has a search function to find cases by ID number or by any of the other data entries. Clicking a row of the table opens that case for review. The remainder of the data display area consists of PNGs showing images and segmentations. Below the table is a three‐plane display of the three annotations needed for our project: a brain mask, a tumor segmentation, and a CSF ROI. This gives an overview of the data and screens for outliers. On the right of the data table are axial and sagittal views of each image (T1‐weighted, T1‐weighted with Contrast, T2‐weighted, and fluid‐attenuated inversion recovery [FLAIR]) with the brain mask overlaid. These are corresponding slices on each co‐registered image, allowing the registration to be visually confirmed, see Figure 2a for example. Below those pictures is a snapshot of the CSF ROI to confirm that it is indeed in the CSF on all images. Below those are scaled density plots of the image intensities within each segmented region. For normalized images, these should fall in an expected range, such as [‐5, 5], with outliers raising suspicion. Finally, the far‐right column visualizes functional images, such as diffusion‐weighted images or synthetic pathology maps.^6^ Instead of overlaid segmentations, a crosshair indicates the maximum intensity voxel inside of the tumor ROI. This shows whether extreme values are being created by an artifact or a true biological signal.

We evaluated the dashboard using two large data sets. Each data set was consecutively reviewed by a single reviewer over several reading sessions. We compiled the results of the review to discover the most common failure modes and what proportion of the processed data was acceptable.

First, we used a historic data set of 1380 glioma patients from a single institution. Preoperative brain MR imaging (MRI) studies were downloaded from the picture archiving and communication system and processed, including co‐registration,^7^ brain,^8^ and cerebrospinal fluid^9^ segmentation, and intensity normalization using open‐source software packages. Tumors were segmented automatically using a deep learning model from the NVIDIA‐CLARA platform pre‐trained on BraTS data.‡ A processing failure was noted only if the upstream processing succeeded. For instance, if a tumor segmentation failed because of bad image registration, only the registration failure was noted. If two failures were judged to have occurred independently, both were noted. Second, we reviewed the 2018 MICCAI BraTS data (285 patients).^10^, ^11^, ^12^ These data were already skull‐stripped and co‐registered, and ground‐truth tumor segmentation was provided for each study.

## 3 RESULTS

The review of all 1665 studies was completed by a single reader (5 years of experience) at an average pace of 100 studies/h. In the authors’ experience, reviewing the same data manually using standard open‐source data viewers (Insight toolkit ITK‐SNAP^13^) takes about 6 min per case or 10 cases/h. Thus, the dashboard provided 10 times faster data review.

The resulting review logs were used to categorize each study as acceptable, acceptable with minor errors, or unacceptable because of poor data quality (e.g., artifact) or data processing failures. The numbers of cases of each quality are listed in Table 1. The most common failure modes in our data were automatic tumor segmentation, with a 9.6% failure rate, and image registration, with a 4.6% failure rate. For data from our institution, 1181 of 1380 (86%) studies were of acceptable quality. For the BraTS data, 242 of 285 (85%) were acceptable: 219 had no errors, 23 had minor errors, and 43 had unacceptable data quality because of our data processing failures (*n *= 29) or raw image quality (*n *= 14). These 14 cases primarily had excessively cropped fields of view that overlapped with ground truth tumor segmentations. Some examples of this are shown in Figure 2b. Without careful data review, these would have gone unnoticed in the subsequent data analysis.

**TABLE 1:** Results of data review with specific review criteria highlighted. Base image quality and normalized intensity ranges are NA since they were only evaluated as acceptable or unacceptable (failure). The combined review result considers all review categories to assign an overall data quality. (a) Results for 1380 clinical studies from our institution. (b) Results for 285 clinical studies from the 2018 Brain Tumor Segmentation Challenge

(a) Clinical data	Acceptable	Minor errors	Failure
Image quality	1360	NA	20
Brain mask	1166	184	30
Registration	1261	55	64
Tumor segmentation	1193	54	133
CSF localization	1228	128	24
Normalization	1350	NA	30
Review result	888	293	199

(b) BraTS data	Acceptable	Minor errors	Failure
Image quality	269	NA	16
Brain mask	266	19	0
Registration	283	1	1
Tumor segmentation	285	0	0
CSF localization	265	15	5
Normalization	261	NA	24
Review result	219	23	43

**FIGURE 3:**
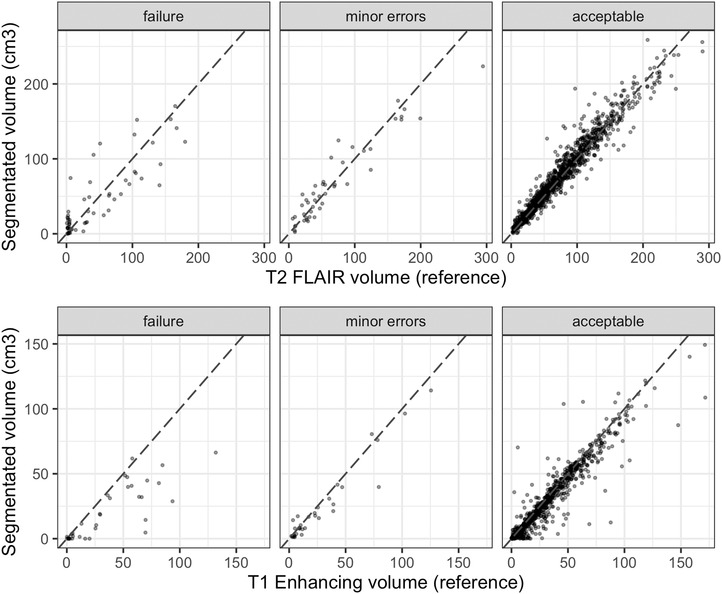
Segmented tumor volume versus reference tumor volume for various levels of data quality. Top: Total T2‐ fluid‐attenuated inversion recovery (FLAIR) hyperintensity volume. Bottom: T1‐enhancing volume. The dashed line indicates agreement.

As an example of the benefit of data review, we calculated the error in segmented tumor volume for each level of data quality (acceptable, minor errors, and failure) on the data from our institution using reference tumor volumes from previous clinical research studies. For this, we only used cases that had acceptable image quality, registrations, and brain masks so that the tumor segmentation was the deciding factor in the data quality. As expected, better quality segmentations had a smaller average error in tumor volume. This was the case for both total tumor volume (T2‐FLAIR hyperintensity) and T1‐enhancing volume (Table 2). The acceptable data had an average total volume error of 12.2 cm^3^ which was 55% smaller than the average error for failed segmentations (27.4 cm^3^) and 40% smaller than the average error in segmentations with minor inaccuracies (20.5 cm^3^). Failed segmentations had 276% higher average error in enhancing volume than segmentations with acceptable quality or minor errors (25.7 cm^3^ vs. 9.3 cm^3^). Figure 3 shows the agreement in tumor volumes for the specific cases. Some outliers in the acceptable data are due to low‐contrast lesions that would likely have high human reader variability as well. Overall, these results show that studies that pass review are in better agreement with ground truth.

**TABLE 2:** Root‐mean‐square‐error (RMSE) in segmented tumor volume for segmentations with varying levels of quality

**Data quality**	***N***	**T2‐FL RMSE (cm^3^)**	**T1‐EV RMSE (cm^3^)**
Acceptable	1192	12.2	9.3
Minor errors	47	20.5	9.3
Failure	65	27.4	25.7

## 4 DISCUSSION

We implemented a lightweight, efficient review dashboard for MRI data sets. The dashboard enabled a complete, structured, human review of data quality of 1380 studies from our institution and 285 publicly available studies. The data review procedure provided useful information on the failure rates in various parts of our data processing pipeline and uncovered possibly problematic images in the BraTS challenge data.

There are published tools for graphically displaying imaging data with the goal of assessing quality.^14^, ^15^ Specifically, these tools evaluate the quality of the raw images themselves through visual inspection or quantitative metrics. While the high underlying image quality is important, it does not guarantee the accuracy of derived data, such as registrations or segmentations. Our dashboard allows the inspection of the images themselves, as well as these other products, to verify the end‐to‐end success of the data processing pipeline. This improves confidence in results stemming from the analysis of the processed imaging data.

Dashboards are not the only way to identify unwanted outliers in imaging data sets. Deep‐learning‐based segmentation models can provide their own uncertainty measurements, which can help identify low‐quality data.^16^ Another approach is to use image features and machine learning to learn to identify failures. However, like the results we observed (Figure 3), even seemingly obvious features such as reference volume measurements are not necessarily perfectly‐correlated with data quality. When analyzing a new data set or processing scheme, the best features are generally not known beforehand. This creates a “chicken‐and‐the‐egg” problem, where training an automatic detection algorithm relies on having (manually) reviewed data to begin with. However, if the data has already been reviewed, the automatic algorithm is unnecessary. Additionally, with an automatic algorithm, some amount of inaccuracy is inevitable. This is especially true if it was developed using data other than the data being currently evaluated. The strength of a data review dashboard is that it enables fast human review without any prior knowledge of the failure modes in the data set. The entire data set can be reviewed one time to confidently establish quality levels without the extra resources to develop, implement, and validate automated methods.

In addition to its many benefits, the dashboard approach has a few limitations, primarily related to the trade‐off between review speed and completeness. We chose fixed, pre‐rendered two‐dimensional PNG images that displayed key informative slices for each image. This reduced amount of data takes less time for a human to review while still enabling quality assessment. The limitation is that artifacts or segmentation errors may not be visible on those key slices. However, we felt that our desired level of data review—acceptable, minor errors, or failure—did not require a substantial increase in review time and complexity. The other limitation is that the imaging data cannot be edited inside the dashboard and any small errors must be handled in separate software. However, we found that existing tools for editing segmentations by hand were far superior.

## 5 CONCLUSION

We developed a lightweight, efficient, easy‐to‐use data review tool to evaluate imaging data sets. It allows the confident evaluation of the quality of a very large amount of data in a reasonable amount of time. Through our review, we characterized the failure rates in our data processing pipeline and found that higher data quality overall was correlated with smaller volumetric errors. A simplified version of our dashboard that can be used for other MR research data sets is available online.

## AUTHOR CONTRIBUTION

Evan Gates, David Fuentes, and Dawid Schellingerhout conceptualized the project. Evan Gates and Adrian Celaya tested and evaluated the dashboard. Evan Gates collected the final data and analyzed it. Dawid Schellingerhout and Dima Suki provided clinical expertise and supervision for the project. Evan Gates, Adrian Celaya, and David Fuentes drafted the manuscript. All authors reviewed approve of the final manuscript.

## Footnotes

*
https://shiny.rstudio.com/

† Since they are specific to the research project that this dashboard was designed for, these plots are not included in the publicly available version of the dashboard.

‡
https://developer.nvidia.com/clara‐medical‐imaging
